# Increased Mortality Rates Caused by Highly Pathogenic Avian Influenza Virus in a Migratory Raptor

**DOI:** 10.1002/ece3.71715

**Published:** 2025-07-06

**Authors:** Neil Paprocki, Jeff Kidd, Courtney J. Conway

**Affiliations:** ^1^ Idaho Cooperative Fish and Wildlife Research Unit University of Idaho Moscow Idaho USA; ^2^ Kidd Biological, Inc. Anacortes Washington USA; ^3^ U.S. Geological Survey, Idaho Cooperative Fish and Wildlife Research Unit University of Idaho Moscow Idaho USA

**Keywords:** avian flu, *Buteo lagopus*, infectious disease, movement ecology, population decline, survival

## Abstract

Highly pathogenic avian influenza virus (HPAIV) has caused extensive mortalities in wild birds with a disproportionate impact on raptors since 2021. The population‐level impact of HPAIV can be informed by telemetry studies that track large samples of initially healthy, wild birds. We leveraged movement data from 71 rough‐legged hawks ( *Buteo lagopus* ) across all major North American migratory bird flyways concurrent with the 2022–2023 HPAIV outbreak and identified a total of 29 mortalities, of which 11 were confirmed, and an additional ~9 were estimated to have been caused by HPAIV. We estimated a 28% HPAIV cause‐specific mortality rate among rough‐legged hawks during a single year concurrent with the HPAIV outbreak in North America. Additionally, the overall mortality rate during the HPAIV outbreak (47%) was significantly higher than baseline annual mortality rates (3%–17%) suggesting that HPAIV‐caused deaths were additive above baseline mortality levels. HPAIV mortalities were concentrated within the Central and Atlantic flyways during prebreeding migration and peaked in April 2022 when large‐scale HPAIV mortalities were reported in other wild birds throughout North America. HPAIV exposure was most likely caused by scavenging or preying on infected waterfowl, as rough‐legged hawks are known to opportunistically scavenge during the nonbreeding season. We utilized movement data to identify a continental‐scale HPAIV cause‐specific mortality event in rough‐legged hawks that has the potential to exacerbate ongoing population declines. Our study highlights the usefulness of monitoring movement data to pinpoint sources of mortality that can help better understand the drivers of population change, even if studies are focused on other research questions.

## Graphical abstract

We utilized movement data to identify a continental‐scale mortality event caused by highly pathogenic avian influenza virus (HPAIV) in a single species of migratory bird, the rough‐legged hawk ( *Buteo lagopus* ). We estimated an HPAIV cause‐specific annual mortality rate of 28% that substantially elevated the overall annual mortality rate (47%) above baseline levels (3%–17%) and has the potential to exacerbate ongoing population declines.

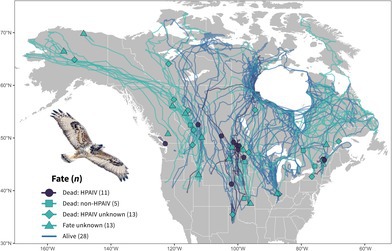

## 1 Introduction

The extent and ubiquity of global bird population declines is becoming increasingly concerning (Inger et al. 2015; Rosenberg et al. 2019). Failure to identify the cause(s) of population declines (e.g., McClure et al. 2017) is one of many reasons why declines in avifauna persist. Survival is a key vital rate that drives population growth rates, especially in longer‐lived species with slower life histories (Clark and Martin 2007; Saether and Bakke 2000). Consequently, identifying cause‐specific sources of mortality is critical to understanding the drivers of population change (e.g., Goldstein et al. 1996). One emerging cause of avian mortality is highly pathogenic avian influenza virus (HPAIV) subtype H5, particularly clade 2.3.4.4, which has caused extensive mortalities in wild birds globally since 2014 (Wille and Barr 2022). In December 2021, a strain of HPAIV (the H5N1 clade 2.3.4.4b) was detected in eastern Canada after causing widespread mortalities in European wild birds during the spring of 2021 (Caliendo et al. 2022; European Food Safety Authority et al. 2023). The virus quickly spread to the United States and has caused at least tens of thousands of wild bird mortalities throughout North America since December 2021 (Avery‐Gomm et al. 2024; Giacinti et al. 2024; USDA APHIS 2024). Water‐associated birds (orders Anseriformes, Charadriiformes, Suliformes) have been the predominant assemblage of wild birds affected by HPAIV, but raptors (*sensu* McClure et al. 2019) have also been disproportionately impacted since 2021 (Avery‐Gomm et al. 2024; European Food Safety Authority et al. 2023; Giacinti et al. 2024; Yang et al. 2024).

In Europe, over 1000 individual raptors represented 12% of all wild bird HPAIV detections from 2020 to 2023 (European Food Safety Authority et al. 2023). In Canada, raptors (especially Accipitriformes and Strigiformes) had some of the highest HPAIV detection rates and accounted for 26% (*N* = 439 of 1710) of all wild bird detections from November 2021 to November 2022 (Giacinti et al. 2024). Larger‐bodied raptors seem to be disproportionately affected by HPAIV (Giacinti et al. 2024; Günther et al. 2024; Hall et al. 2024) with studies reporting relatively higher HPAIV detections among raptors of the following species: (1) white‐tailed sea eagle ( *Haliaeetus albicilla* ), common buzzard ( *Buteo buteo* ), and other unidentified *Buteo* species (Germany; Günther et al. 2024); and (2) great horned owl ( *Bubo virginianus* ), bald eagle ( *Haliaeetus leucocephalus* ), red‐tailed hawk ( *Buteo jamaicensis* ), and peregrine falcon ( *Falco peregrinus* ; Minnesota, USA; Hall et al. 2024). Preying or scavenging on infected avian prey has been empirically demonstrated to transmit HPAIV to raptors (Bertran et al. 2012). Consequently, a leading hypothesis to explain why larger raptors are disproportionately infected with HPAIV is that larger raptors are more likely to prey or scavenge on other infected avian species that also tend to be larger‐bodied (e.g., geese, ducks; Giacinti et al. 2024; Günther et al. 2024; Nemeth et al. 2023).

One source of HPAIV reporting comes from the opportunistic sampling of sick or dead birds that are admitted to wildlife rehabilitation centers (WRC; Caliendo et al. 2022; Giacinti et al. 2024; Hall et al. 2024). HPAIV and other pathogen reporting from WRC is useful for general pathogen surveillance but inferring population‐level impacts from such reporting can be challenging, for one, because absence data are often lacking or biased (e.g., absences reported from already sick or dead birds, birds submitted closer to population centers; Camacho et al. 2016; Hall et al. 2024). Survey data from routine surveillance activities of wild birds (e.g., live‐trapping, band resighting, breeding colony and nesting surveys) are more amenable to inferring population changes and such data have suggested population‐level impacts from HPAIV in some species (Avery‐Gomm et al. 2024; Nemeth et al. 2023; Tremlett et al. 2025). The remote monitoring of bird movements via animal borne tracking technology (hereafter “GPS‐tracking”) also allows for pathogen surveillance if mortalities are identified quickly, and bird carcasses subsequently retrieved and tested. GPS‐tracking and subsequent carcass retrieval represents an intriguing approach to assessing the population‐level impacts of pathogens, particularly if large sample can be obtained, because the proportion of affected versus unaffected individuals can be quantified (e.g., Duriez et al. 2023). Additionally, GPS‐tracking and subsequent carcass retrieval provides increased spatial and temporal resolution of cause‐specific mortalities from initially healthy, wild birds (Serratosa et al. 2024). GPS‐tracking data also facilitate an understanding of the potential spread of HPAIV and other pathogens via seasonal bird migration, which has been identified as the primary driver of the spread of HPAIV (Yang et al. 2024).

In this study, we utilized movement and mortality data from GPS transmitters attached to 71 rough‐legged hawks ( *Buteo lagopus* ) that occurred concurrently with the 2022–2023 outbreak of HPAIV in North America to assess the population‐level impact on the species. The rough‐legged hawk breeds throughout arctic and subarctic regions of the world and performs a complete migration (i.e., all individuals migrate) to overwintering destinations: southern Canada and the contiguous United States for North American populations (Bechard et al. 2020). Rough‐legged hawks primarily eat small mammals but also scavenge, particularly during the non‐breeding season (Paprocki et al. 2024), which could lead to increased susceptibility to HPAIV if birds scavenge on HPAIV‐infected carcasses. Our objectives were to leverage continent‐wide rough‐legged hawk movement and mortality data to (1) document spatial, temporal, and cause‐specific mortality patterns concurrent with the 2022–2023 HPAIV outbreak in North America; (2) quantify estimates of population‐level cause‐specific mortality rates from HPAIV; and (3) determine if HPAIV mortality rates were additive above baseline levels of mortality.

## 2 Materials and Methods

We collected movement data on rough‐legged hawks over a 12‐month period (1 March 2022 to 28 February 2023) that coincided with the initial HPAIV panzootic in North America and during which our study population experienced mortalities from HPAIV (Table 1). We trapped and attached GPS‐Argos (22–30 g; Microwave Telemetry and GeoTrak Inc.) or GPS‐GSM (21–25 g; Ecotone Telemetry or Ornitela) transmitters to 71 rough‐legged hawks that provided location data from 1 March 2022 to 28 February 2023 (hereafter referred to as “study period”). Hawks that provided location data during the study period were captured from 2014 to 2023 across 19 U.S. states and Canadian provinces throughout the species entire North American distribution as part of a large‐scale, ongoing research project. We deployed transmitters on hawks during the following seasons: winter (*n* = 62), breeding (*n* = 4), and migration (*n* = 5). We used standardized high‐resolution photographs taken at capture to age hawks using cycle‐based terminology (Liguori et al. 2020; Robinson et al. 2024): 1C (first cycle), 2C (second cycle), 3C/A2C (third cycle, including after second cycle), or A3C (after third cycle). To determine age at time of death (Table 1), we forward calculated from age at capture using 1 September as the annual transition date between molt cycles based on the species average molt timing (Bechard et al. 2020). We categorized the sex of each hawk based on either DNA (*n* = 65) or morphometrics (*n* = 6). We collected feathers, blood (drawn from brachial vein), or cheek swabs from a subset of hawks to genetically sex individuals (Pitzer et al. 2008) and used morphometrics from hawks sexed genetically to assign sex to those not genetically sexed (Tomalty et al. 2016) with a success rate of 100% based on either body mass or wing length.

**TABLE 1:** Summary of 29 rough‐legged hawk mortalities during an outbreak of highly pathogenic avian influenza virus (HPAIV) in North America from 1 March 2022 to 28 February 2023.

Sex	Age^a^	Mortality date	Fate	Cause of death	HPAIV PCR result	Mortality flyway
M	A4C	22 March 2022	Confirmed dead	Unknown	Not tested	Central
M	2C	23 March 2022	Confirmed dead	HPAIV	Positive	Central
F	2C	5 April 2022	Confirmed dead	HPAIV	Positive	Central
F	A4C	16 April 2022	Likely dead	Unknown	Not tested	Central
F	A3C	16 April 2022	Confirmed dead	HPAIV	Positive	Atlantic
F	1C	17 April 2022	Confirmed dead	HPAIV	Positive	Atlantic
M	A5C	22 April 2022	Confirmed dead	Unknown/non‐HPAIV	Negative	Central
F	1C	22 April 2022	Confirmed dead	HPAIV	Positive	Mississippi
F	A5C	23 April 2022	Confirmed dead	HPAIV	Positive	Central
M	A3C	24 April 2022	Confirmed dead	HPAIV	Positive	Central
F	A4C	24 April 2022	Confirmed dead	HPAIV	Positive	Central
M	A3C	26 April 2022	Confirmed dead	HPAIV	Positive	Central
F	A4C	12 May 2022	Likely dead	Unknown	Not tested	Pacific
M	1C	13 May 2022	Likely dead	Unknown	Not tested	Mississippi
F	1C	21 June 2022	Confirmed dead	Unknown	Not tested	Atlantic
F	1C	21 June 2022	Confirmed dead	Unknown	Not tested	Atlantic
F	5C	7 October 2022	Likely dead	Unknown	Not tested	Central
F	A6C	8 October 2022	Confirmed dead	Head trauma/non‐HPAIV	Negative	Mississippi
M	A6C	4 November 2022	Likely dead	Unknown	Not tested	Central
F	A4C	10 November 2022	Confirmed dead	Unknown/Possible predation	Not tested	Central
M	A6C	14 November 2022	Likely dead	Unknown	Not tested	Central
M	A4C	6 December 2022	Confirmed dead	Unknown/non‐HPAIV	Negative	Central
M	A4C	15 December 2022	Confirmed dead	Unknown/Possible predation	Not tested	Mississippi
F	A5C	23 December 2022	Confirmed dead	Unknown/Possible predation	Not tested	Pacific
F	2C	31 December 2022	Confirmed dead	HPAIV	Positive	Pacific
F	2C	10 January 2023	Confirmed dead	HPAIV	Positive	Central
F	A4C	20 January 2023	Confirmed dead	Unknown/Possible predation	Not tested	Atlantic
F	2C	22 January 2023	Confirmed dead	Unknown/non‐HPAIV	Negative	Mississippi
M	2C	21 February 2023	Confirmed dead	Gunshot/non‐HPAIV	Not tested^b^	Central

^a^ Age at time of mortality.

^b^ Assumed negative based on cause of death.

We classified fate at the end of the study period into four categories loosely following Buechley et al. (2021): (1) actively functioning transmitter on a moving hawk (“alive”); (2) transmitter data indicated mortality (final GPS locations stationary for > 48 h) but could not be verified in person (“likely dead”); (3) transmitter data indicated mortality and hawk carcass or feather remains found with a transmitter (“confirmed dead”); and (4) offline transmitters on a previously moving hawk (“unknown”). Unknown fates were most often GPS‐GSM transmitters (*n* = 12) that left cell phone service (a common occurrence from late spring to early fall when rough‐legged hawks occupy northern latitudes with very limited cell phone service) and never came back online as well as two GPS‐Argos transmitters that ceased functioning while movement data suggested hawks were still alive. For analysis purposes, we grouped “likely dead” and “confirmed dead” into a single “dead” category because 100% (*n* = 23) of cases where GPS transmitter data indicated a mortality and for which the transmitter was recovered resulted in a “confirmed dead” fate designation (i.e., hawk remains were also found along with the transmitter).

When GPS transmitters suggested a mortality (stationary location for > 48 h), we contacted local authorities who attempted to locate and recover carcasses. If mortalities were located, suitable carcasses were recovered and sent to diagnostic laboratories in the USA and Canada for necropsy and HPAIV testing. All HPAIV testing was conducted using real‐time reverse transcription polymerase chain reaction (RT‐PCR; Spackman et al. 2002). We classified cause of death for hawks that were “confirmed dead” based on a combination of HPAIV testing, necropsy results, or field remains (Table 1). We only attributed cause of death to HPAIV if hawks tested positive via RT‐PCR and assumed HPAIV was the cause of death in all positive RT‐PCR detections as past research has shown 97% of birds testing positive for HPAIV had gross or histological evaluations consistent with HPAIV infection as cause of death (Giacinti et al. 2024). Cause of death was classified as unrelated to HPAIV (“non‐HPAIV”) if (1) HPAIV was not detected via RT‐PCR (*n* = 4) or (2) HPAIV status was not tested (often because a carcass was too necrotic) but necropsy results were consistent with a non‐HPAIV related cause of death (*n* = 1; Table 1). Cause of death for hawks classified as “likely dead” were left as unknown. We assigned all mortality locations to North American migratory bird flyways as depicted in Waller et al. (2018).

**FIGURE 1:**
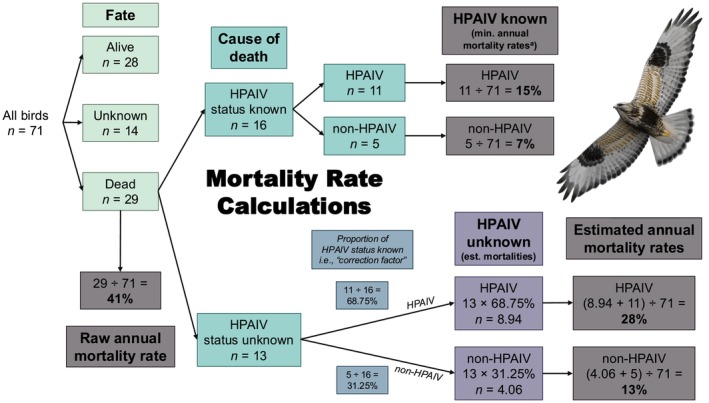
Flow chart detailing the approach used to calculate cause‐specific (HPAIV or non‐HPAIV) and annual mortality rates from a sample of 71 rough‐legged hawks tracked during an outbreak of highly pathogenic avian influenza virus (HPAIV) in North America. Rough‐legged hawk illustration by Bryce W. Robinson. ^a^Minimum annual mortality rates assume none of the “unknown” mortalities were caused by HPAIV.

We used logistic regression via the “glm” function from the “stats” R package (R Core Team 2025) to predict HPAIV fate explained by sex and age class. We tested whether HPAIV mortality risk differed by sex and age class by comparing HPAIV positive birds to all other birds (non‐HPAIV, HPAIV unknown, fate unknown, alive). Due to sample size constraints, we reduced our age classes to two groups: (1) adult (included 2C, 3C/A2C, and A3C age classes); or (2) juvenile (included only the 1C age class). We generated two estimates each of population‐level (1) HPAIV mortality rates, and (2) non‐HPAIV mortality rates. Our detailed approach used to calculate HPAIV mortality rates is shown in Figure 1. Briefly, we calculated minimum population‐level HPAIV and non‐HPAIV mortality rates as the proportion of total birds tracked that were confirmed to have died of either HPAIV or non‐HPAIV causes. We then used the proportion of all HPAIV and non‐HPAIV mortalities (i.e., excluding HPAIV status unknown mortalities) that were HPAIV positive as a correction factor to estimate the number of HPAIV and non‐HPAIV mortalities among our sample of confirmed and likely dead hawks where HPAIV fate was unknown (*n* = 13). Estimated HPAIV and non‐HPAIV mortality rates were then calculated as the proportion of total birds tracked that were either confirmed or estimated to have died of HPAIV or non‐HPAIV causes. Missing birds classified as fate “unknown” may also have died from HPAIV or other causes, however an unknown proportion of this group may have still been alive with nonfunctioning transmitters and we therefore chose to not include them in population‐level mortality estimates to be conservative.

We also classified the fate of all tracked hawks at the end of each of the two previous and one subsequent 12‐month periods (hereafter “baseline periods”) when our study population did not experience confirmed HPAIV deaths (i.e., March 2020–February 2021; March 2021–February 2022; March 2023–February 2024) and during which time we were also tracking hawks across large portions of North America. We then used logistic regression via the “glm” function from the “stats” R package (R Core Team 2025) to predict the fate (1 = “likely dead” or “confirmed dead”; 0 = “alive” or “unknown”) of tracked birds across four, 12‐month periods. We also ran a separate analysis that included only known‐fate birds (i.e., “unknown” fate birds were excluded) to test the robustness of our modeling approach. We included as control variables the day during each 12‐month period when tracking of an individual was initiated (day 1 = 1 March) and transmitter type (either GPS‐GSM or GPS‐Argos). We expected individuals whose tracking was initiated closer to the beginning of each 12‐month period to be more likely to experience a mortality event due to an increase in exposure time. GPS‐GSM transmitters send location data via the cellphone network (while GPS‐Argos transmitters send data via satellites) and individuals in our study system often spend the entire breeding season and portions of migration “offline” because of a lack of reliable cell phone coverage at far northern latitudes. Consequently, we wanted to control for any differences in the proportion of individuals tracked via GPS‐GSM or GPS‐Argos transmitters among years. We expected GPS‐GSM transmitters to have a reduced ability to detect mortalities because of an increase in “unknown” fates caused by mortalities that occurred in areas that lacked cell phone coverage. We then included 12‐month period as a 4‐level factor (3 baseline periods and 1 study period) to determine if mortality rates during our HPAIV study period were additive above baseline mortality rates. We used Tukey pairwise comparisons from the “emmeans” package (Lenth 2023) to determine pairwise differences in mortality rates among years.

## 3 Results

**FIGURE 2:**
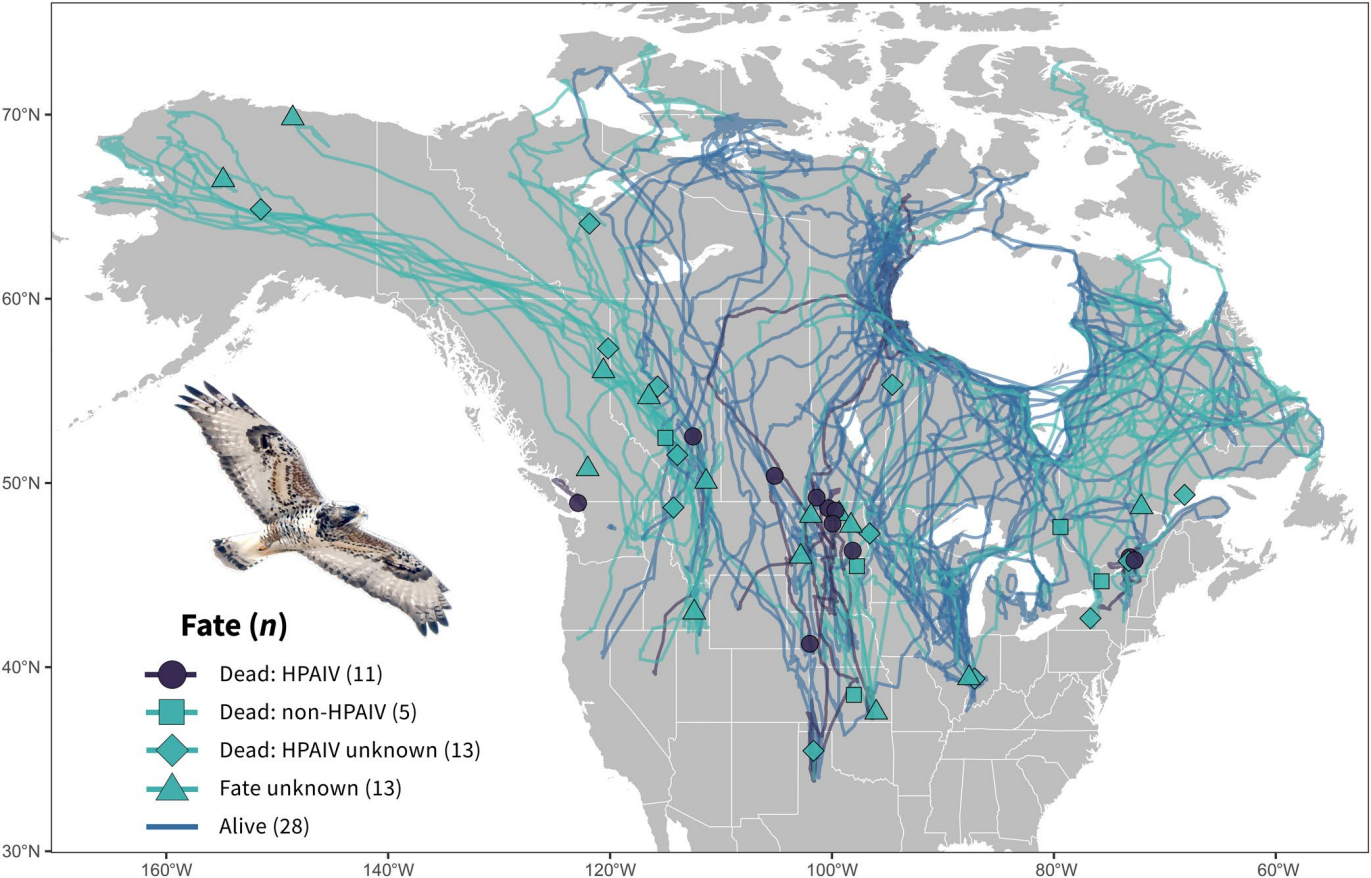
Mortality events (circles, squares, diamonds), last known locations of missing individuals (triangles), and cumulative movements of 71 rough‐legged hawks tracked during an outbreak of highly pathogenic avian influenza virus (HPAIV) in North America from 1 March 2022 to 28 February 2023. Confirmed HPAIV mortalities shown in dark purple. Movements include 28 individuals known to be alive at the end of the study period (blue paths).

**FIGURE 3:**
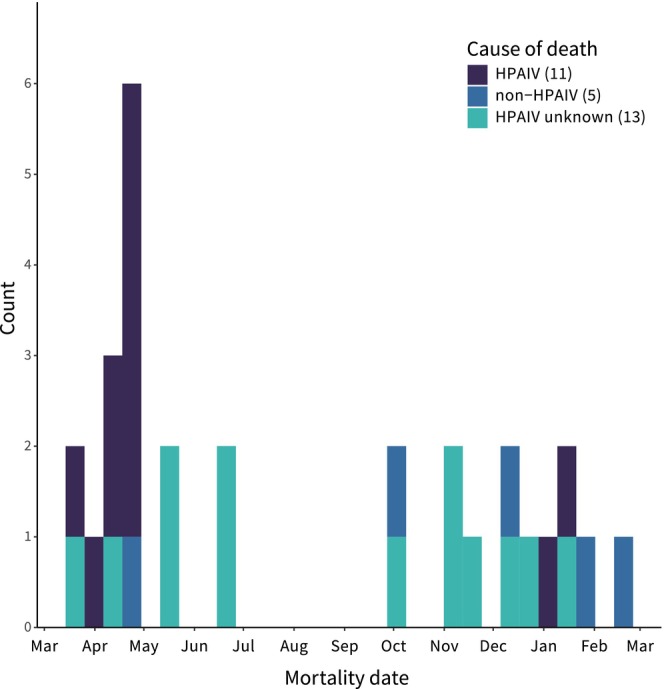
Summary of 29 rough‐legged hawk mortalities by date and cause of death during an outbreak of highly pathogenic avian influenza virus (HPAIV) in North America from 1 March 2022 to 28 February 2023.

We tracked a total of 71 rough‐legged hawks for portions of the 1 year. Study period (1 March 2022 to 28 February 2023) during which hawks experienced HPAIV mortalities (Figure 2; Figure 3). During the study period, we detected a total of 29 mortalities of which 23 were confirmed via carcass or feather remains collected from the field (“confirmed dead”), while the remaining six were confirmed via movement data only (“likely dead”; Table 1). During the study period, we detected (1) 11 mortalities caused by HPAIV; (2) 5 mortalities unrelated to HPAIV (“non‐HPAIV”); and (3) 13 mortalities where we could not determine if HPAIV was related to death (“HPAIV status unknown”). Cause of death for non‐HPAIV mortalities was attributed to persecution/gunshot (*n* = 1), head trauma (*n* = 1), or was unknown (*n* = 3; Table 1). Reasons for mortalities not being tested for HPAIV included inaccessible mortalities (*n* = 6) or carcass decomposition/feather remains that precluded testing (*n* = 8). We detected HPAIV mortalities in all four major North American migratory flyways (Table 1), but mortalities were more heavily concentrated in the Central flyway (*n* = 7 of 11; Figure 2; Table 1). We detected fewer HPAIV mortalities in the Atlantic (*n* = 2), Mississippi (*n* = 1), and Pacific (*n* = 1) flyways. HPAIV mortalities were first detected on 23 March 2022 and reached their peak in April 2022 (Figure 3). We also detected a smaller peak in HPAIV mortalities in late December 2022 and early January 2023 (Figure 3). We did not detect an age (juvenile 𝛽 = 0.558; SE = 0.773; *p* = 0.470) or sex (male 𝛽 = −0.951; SE = 0.728; *p* = 0.191) bias among HPAIV mortalities compared to all tracked birds in our sample.

**FIGURE 4:**
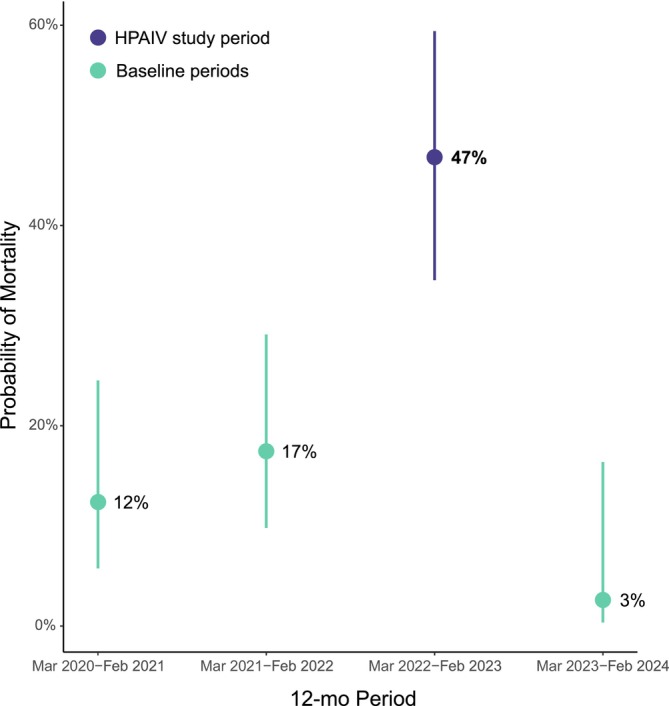
Rough‐legged hawk annual mortality rate was higher during an outbreak of highly pathogenic avian influenza virus (HPAIV) in North America compared to three baseline periods. Points (group means) and whiskers (95% confidence intervals) are from a generalized linear model predicting fate after controlling for number of days tracked and transmitter type.

We found a minimum population‐level HPAIV mortality rate of 15% based on all confirmed HPAIV deaths within our total sample (Figure 1). We estimated an additional 8.94 hawks from our sample died of HPAIV by applying an HPAIV correction factor to our sample of confirmed and likely dead hawks for which HPAIV fate was unknown (Figure 1). We then found an estimated population‐level HPAIV mortality rate of 28% based on all confirmed and estimated HPAIV deaths within our total sample (Figure 1). In contrast, minimum and estimated population‐level mortality rates from non‐HPAIV causes were 7% and 13%, respectively, and were more similar to annual mortality rates from baseline periods when we did not detect mortalities caused by HPAIV (Figure 1; Figure 4).

After controlling for the effects of tracking start day within a 12‐month period (𝛽 = −0.00135; SE = 0.00146; *p* = 0.355) and transmitter type (GPS‐Argos 𝛽 = 1.14; SE = 0.366; *p* = 0.00194), we found that 12‐month period explained rough‐legged hawk fate (likelihood‐ratio 𝜒^2^ = 32.8, df = 3, *p* < 0.0001) such that the annual mortality rate during the HPAIV study period was significantly higher compared to all three baseline periods based on Tukey pairwise comparisons (Table 2; Figure 4). Our results were similar when we included only known‐fate bird‐years (i.e., excluding *n* = 43 unknown fates): after controlling for the effects of tracking start day within a 12‐month period (𝛽 = −0.00244; SE = 0.00148; *p* = 0.0983) and transmitter type (GPS‐Argos 𝛽 = 0.912; SE = 0.386; *p* = 0.0181), we still found that 12‐month period explained rough‐legged hawk fate (likelihood‐ratio 𝜒^2^ = 34.3, df = 3, *p* < 0.0001) such that the annual mortality rate during the HPAIV study period was significantly higher compared to all three baseline periods based on Tukey pairwise comparisons (*p* < 0.005 for all three pairwise comparisons with the HPAIV study period). However, nearly all annual mortality rates were higher after the exclusion of “unknown” fates (known‐fate annual mortality estimates: 2020/21 = 16%; 2021/22 = 23%; 2022/23 = 57%; 2023/24 = 3%; compare with Figure 4).

**TABLE 2:** Tukey pairwise comparisons of rough‐legged hawk fate among 12‐month periods after controlling for the effects of tracking start date and transmitter type.

Contrast	Estimate	SE	df	z.ratio	*p*
Year 2020/21–year2021/22	−0.41	0.498	Inf	−0.814	0.8477
**Year 2020/21**–**year 2022/23**	**−1.83**	**0.499**	**Inf**	**−3.668**	**0.0014**
Year 2020/21–year 2023/24	1.68	1.909	Inf	1.541	0.4131
**Year 2021/22**–**year 2022/23**	**−1.43**	**0.421**	**Inf**	**−3.390**	**0.0039**
Year 2021/22–year 2023/24	2.08	1.060	Inf	1.960	0.2033
**Year 2022/23**–**year 2023/24**	**3.51**	**1.050**	**Inf**	**3.327**	**0.0049**

*Note:* Pairwise comparisons with *p* values < 0.05 are shown in bold.

## 4 Discussion

We documented an alarming 28% population‐level mortality rate among North American rough‐legged hawks caused by highly pathogenic avian influenza virus (HPAIV) during a one‐year period between 2022 and 2023. Our population‐level HPAIV mortality rates were considerably higher than concurrent non‐HPAIV mortality rates and were based on the GPS tracking of 71 individual rough‐legged hawks throughout their entire North American distribution. Moreover, the annual mortality rate during the HPAIV outbreak (47%) was significantly higher than annual mortality rates across three baseline years (3%–17%) suggesting that HPAIV mortalities were additive above baseline levels. A substantial peak in rough‐legged hawk HPAIV mortalities occurred in April 2022, during which time hawks throughout North America were in the early stages of northward pre‐breeding migration. Concurrent with rough‐legged hawk pre‐breeding migration in April 2022, large‐scale HPAIV mortality events were occurring in other wild birds throughout North America, but particularly among migrating waterfowl within the Central and Atlantic flyways (Giacinti et al. 2024) where the majority of our confirmed HPAIV mortalities occurred.

While HPAIV exposure routes were not explicitly known in our study system, a leading exposure‐route hypothesis is the preying or scavenging on HPAIV‐infected waterfowl carcasses. We can point to at least four lines of evidence that support exposure via preying or scavenging on infected waterfowl carcasses. First, at least one rough‐legged hawk mortality that tested positive for HPAIV in the Central flyway was found in the immediate vicinity of a dead snow goose ( *Anser caerulescens* ) carcass. Second, rough‐legged hawks have been empirically shown to predate or scavenge waterfowl during winter (1.7% of overall prey occurrences from eDNA; Paprocki et al. 2024) and the species is well‐known for opportunistic scavenging in general (Bechard et al. 2020; Paprocki et al. 2024). Third, our observed peak in HPAIV mortalities occurred at the same time, and within the same flyways, as major waterfowl mortality events that coincided with peak goose migration (Fink et al. 2023; Giacinti et al. 2024). Lastly, feeding on infected avian prey has been empirically demonstrated to transmit HPAIV to raptors (Bertran et al. 2012). Overall, these lines of evidence suggest that a high degree of spatial and temporal overlap between both migrating rough‐legged hawk and wild goose populations, and large‐scale HPAIV mortality events among waterfowl in the spring of 2022, coupled with a predisposition for opportunistic scavenging created a “perfect storm” of events that led to a large‐scale HPAIV mortality event among rough‐legged hawks. Alternative exposure‐route hypotheses include (1) air‐borne transmission (Bertran et al. 2017) via proximity to, but not consumption of, HPAIV‐infected waterfowl carcasses, or (2) mammalian‐to‐bird transmission (Caserta et al. 2024) via consumption of infected mammalian prey given that rough‐legged hawks primarily consume mammalian prey captured on the ground (e.g., ground‐dwelling small mammals; Paprocki et al. 2024).

We suspect that several rough‐legged hawk mortalities whose HPAIV status was unknown were indeed caused by HPAIV. At least four other mortalities of unknown cause occurred from March to May 2022 in conjunction with waterfowl migration (Fink et al. 2023) at various locations along migratory flyways from Texas to Alaska. Suspected HPAIV deaths included two Central flyway mortalities on 22 March 2022 and 16 April 2022 from Texas, USA, and North Dakota, USA, respectively. Suspected HPAIV deaths also included Pacific and Mississippi flyway mortalities on 12 May 2022 and 13 May 2022 from Alaska, USA, and Manitoba, Canada, respectively, that both occurred near small bodies of water where migratory waterfowl could have concentrated. Additionally, two mortalities of unknown cause that occurred around the Gulf of St. Lawrence, Quebec, Canada, in late June 2022 were notable for two reasons. First, they both were juvenile hawks that exhibited atypical seasonal movements just prior to death; both conducted 1000–1200 km reverse migrations, flying back south and away from the arctic breeding season range of rough‐legged hawks. Second, both juvenile hawk mortalities occurred simultaneously with a major HPAIV outbreak along the Gulf of St. Lawrence among breeding colonies of northern gannet ( *Morus bassanus* ), common eider ( *Somateria mollissima* ), and common murre ( *Uria aalge* ; Giacinti et al. 2024). While we could not confirm the HPAIV status of unknown mortalities due to inaccessible or decomposed carcasses, our estimated HPAIV mortality rate of 28% accounted for an additional 8.94 hawks from our sample of HPAIV unknown status that may have died of HPAIV.

An important limitation of our dataset was that we may not have been tracking rough‐legged hawks in proportion to their relative regional abundance. If HPAIV mortality risk varied spatially and hawks were not sampled in proportion to their relative abundance, then mortality estimates could be biased high or low depending on the degree of spatial overlap between our sampled population and HPAIV mortality risk. However, most hawks tracked during our study period wintered in either the Pacific or Central flyways (61%; *n* = 43 of 71) where rough‐legged hawk relative winter abundance has been historically greatest (Bechard et al. 2020; Fink et al. 2023) suggesting that we were broadly tracking individuals in proportion to their flyway‐level relative abundance and that our estimates of HPAIV mortality may broadly represent actual continent‐wide mortality rates. Additionally, the HPAIV mortality rates we report for rough‐legged hawks are at the high end of those reported from other GPS‐tracking (maximum of 9.7% in griffon vulture *Gyps fulvus* ; Duriez et al. 2023) or census‐based studies (7.0%–18.7% in common eider, minimum 11.5% in northern gannet; Avery‐Gomm et al. 2024). However, our study is novel in that it represents one of the most geographically comprehensive assessments of HPAIV mortality rates within a single species from an initial sample of healthy, wild birds.

Large‐scale mortality events can cause immediate and potentially irreversible reductions to avian populations (Renner et al. 2024). However, it is not yet known whether our observed rough‐legged hawk HPAIV mortality event suppressed populations enough to be detected by continent‐wide population monitoring programs such as the National Audubon Society's Christmas Bird Count (Paprocki et al. 2014) or eBird (Fink et al. 2023). Additionally, large‐scale mortality events may have a compounding effect on populations if they occur in conjunction with reduced breeding productivity as suggested by previous studies on HPAIV effects on raptors (Duriez et al. 2023; Nemeth et al. 2023). While the rough‐legged hawk's IUCN Red List threat status is Least Concern globally (BirdLife International 2024), populations in North America may have been declining prior to the 2022–2023 HPAIV outbreak (Fink et al. 2023; Oleyar et al. 2021; Paprocki et al. 2014). In North America, it is still unclear to what degree observed population declines were explained by actual population changes or northward distribution shifts to the species overwinter range in relation to survey coverage (Paprocki et al. 2014), but evidence is mounting that rough‐legged hawk populations in North America are indeed declining. The species has been given the highest conservation priority status (“high research and conservation priority”) in the most recent Raptor Population Index (Oleyar et al. 2021) based on long‐term declines observed from both migration and wintering counts. Additionally, short‐term population trends from wintering eBird data in North America show a 17.2% decline from 2011 to 2021 (Fink et al. 2023). It would be helpful for future studies assessing the population status of rough‐legged hawks to quantify how the HPAIV outbreak may have exacerbated pre‐HPAIV population declines given that other studies have found declines in count‐based population indices in species impacted by HPAIV (Tremlett et al. 2025).

Cause‐specific mortalities revealed by remotely monitored movement data provide a unique ability to learn where, when, and potentially how birds die and can help identify potential drivers of population change (Oppel et al. 2021; Serratosa et al. 2024) such as disease outbreaks. We leveraged a continent‐wide GPS‐tracking dataset from a widespread raptor to assess the population‐level impacts of highly pathogenic avian influenza (HPAIV) and found evidence for a significant mortality event that has the potential to exacerbate ongoing population declines. Understanding spatial and temporal patterns in survival rates and cause‐specific mortality via movement data is a burgeoning field (e.g., Buechley et al. 2021; Sergio et al. 2019; Serratosa et al. 2024) and useful next steps to advance our understanding of population declines include: (1) devoting resources to monitoring mortalities and determining causes of death, even if studies are focused on other research questions; and (2) either publishing or incorporating mortality results into population status assessments (e.g., McCabe et al. 2024).

## Author Contributions

**Neil Paprocki:** conceptualization (equal), data curation (lead), formal analysis (lead), funding acquisition (equal), investigation (equal), methodology (lead), project administration (supporting), resources (supporting), software (lead), validation (lead), visualization (lead), writing – original draft (lead), writing – review and editing (equal). **Jeff Kidd:** conceptualization (equal), data curation (supporting), funding acquisition (equal), investigation (equal), methodology (supporting), project administration (equal), resources (equal), supervision (equal), validation (equal), writing – review and editing (equal). **Courtney J. Conway:** conceptualization (equal), funding acquisition (equal), investigation (equal), methodology (equal), project administration (equal), resources (equal), supervision (equal), validation (equal), writing – review and editing (equal).

## Conflicts of Interest

The authors declare no conflicts of interest.

## Data Availability

Data are provided for peer review through Dryad: https://doi.org/10.5061/dryad.n2z34tn92.
